# Comparing Inpatient Complication Rates between Octogenarians and Nonagenarians following Primary and Revision Total Knee Arthroplasty in a Nationally Representative Sample, 2010–2014

**DOI:** 10.3390/geriatrics4010003

**Published:** 2018-12-22

**Authors:** Eric L Smith, Evan M Dugdale, David Tybor, Michael Kain

**Affiliations:** 1 Boston Medical Center, Department of Orthopaedic Surgery, Boston, MA 02118, USA; michael.kain@bmc.org; 2 Boston University School of Medicine, Boston, MA 02118, USA; edugdale@bu.edu; 3 Department of Public Health and Community Medicine, Tufts University School of Medicine, Boston, MA 02111, USA; david.tybor@tufts.edu

**Keywords:** total knee arthroplasty, total knee revision arthroplasty, arthroplasty in octogenarians, arthroplasty in nonagenarians, primary total knee arthroplasty complications, revision total knee arthroplasty complications

## Abstract

We compared the inpatient postoperative complication rates between octogenarians and nonagenarians undergoing primary and revision total knee arthroplasty (TKA). We used the Nationwide Inpatient Sample (NIS) to analyze inpatient admission data from 2010–2014. We compared the rates at which nonagenarians and octogenarians developed each complication following both primary TKA (PTKA) and revision TKA (RTKA). A national estimate of 324,933 patients were included in our study. A total of 313,299 (96.42%) were octogenarians, and 11,634 (3.58%) were nonagenarians. 294,462 (90.62%) underwent PTKA, and 30,471 (9.38%) underwent RTKA. Nonagenarians undergoing PTKA had a higher inpatient mortality rate, and developed sepsis more frequently than octogenarians. Nonagenarians undergoing RTKA had a higher inpatient mortality rate, and developed cardiogenic shock more frequently than octogenarians. In both PTKA and RTKA, nonagenarians received transfusions more frequently, and developed urinary tract infection and acute kidney injury more frequently than octogenarians. In both PTKA and RTKA, nonagenarians sustained a higher inpatient mortality rate than octogenarians. Orthopedic surgeons should counsel nonagenarian patients undergoing both PTKA and RTKA preoperatively about this increased mortality risk, as well as the increased risks of more minor complications like transfusion, urinary tract infection, and acute kidney injury.

## 1. Introduction

The approximately 49 million Americans over age 65 in 2016 is projected to nearly double to 95 million by 2060, and constitute almost a quarter of the US population [1]. Notably, after almost tripling between 1980 and 2010, the number of Americans age 90 and older is expected to quadruple from 2010 to 2050 [2]. Orthopedic surgeons may be hesitant to operate on nonagenarians, due to the high number of preoperative medical comorbidities expected in this population [3]. However, as this group makes up an increasing proportion of the population, there will be an increasing volume of total knee arthroplasty (TKA) operations demanded by nonagenarians. From 2000 to 2006, the number of TKA procedures performed on patients aged 85 years and older increased by 73% [4]. It is therefore important that orthopedic surgeons are aware of any increased postoperative risks in the nonagenarian subpopulation, so that efforts can be made to mitigate these risks perioperatively, and to communicate these risks to patients preoperatively.

Several studies have evaluated the outcomes of nonagenarians undergoing TKA. Alfonso et al., Joshi et al., and Belmar et al. each described experiences with nonagenarians at a single center. Joshi et al. described only one surgical complication (a wound dehiscence) in 18 patients, and Belmar et al. reported no significant surgical complications in 12 patients [5,6]. Alfonso et al. found the majority of complications among 25 patients to be cardiovascular and genitourinary, and also noted delirium (16% of patients) and transfusion (80% of patients) to occur frequently [7]. These single-center studies provided excellent initial insight into the postoperative course of nonagenarians, but are limited by their small sample sizes.

More recently, Jauregui et al. compared the postoperative outcomes of nonagenarians undergoing TKA to those of patients less than 90 years old, using a significantly larger sample size with the National Surgical Quality Improvement Program (NSQIP) database. Nonagenarians were found to have a significantly higher risk of “minor adverse events” compared to patients less than 90 years old, but no significant difference in risk of “serious adverse events”, in a 30-day postoperative period [8]. By using the NSQIP, this study had a greatly increased sample size compared to the aforementioned single-center studies. However, this study compared nonagenarians against all patients less than age 90, rather than focusing on the incremental risk of nonagenarians compared to a slightly younger cohort. This marginal risk between these two adjacent elderly age groups may be more useful to patients and physicians alike in the decision-making process, when considering surgical intervention.

The purpose of our study was to compare the postoperative inpatient complication rates of nonagenarians against those of octogenarians undergoing primary and revision TKA.

## 2. Materials and Methods

We used the Nationwide Inpatient Sample (NIS) to gather data on both primary and revision TKA procedures performed between 2010 and 2014. The NIS features prospectively collected data from a stratified sample comprising 20% of all community hospital discharges nationwide. NIS-provided sample weights allow for the extrapolation of national estimates from the sample. Currently, 46 states and the District of Columbia contribute to the NIS dataset, which represents approximately 97% of the United States population. We analyzed data from 2010–2014, and used NIS-provided guidelines for aggregating the annual samples [9].

We used ICD-9 codes to isolate patients undergoing the procedures of interest, PTKA and RTKA. Patients with the ICD-9 Procedure Code 81.54 (total knee replacement) were included in the PTKA group. Patients matching 00.80 (revision of knee replacement, total), 00.81 (revision of knee replacement, tibial component), 00.82 (revision of knee replacement, femoral component), 00.83 (revision of knee replacement, patellar component), 00.84 (revision of knee replacement, tibial insert), or 81.55 (revision of knee replacement, not otherwise specified) were included in the RTKA group. Any records matching both ICD-9 codes for primary and revision total knee arthroplasty were excluded from the study.

The NIS provides patients’ ages in years. We used this to generate one treatment group of patients aged 80–89 years, and one treatment group of patients aged 90–99 years.

Patient characteristics and outcomes were obtained by using NIS-provided metrics (e.g., length of stay, total charges, number of chronic conditions, etc.). Patient complications were identified using ICD-9 diagnosis and procedure codes. Specifically, ICD-9 codes suggesting transfusion, deep venous thrombosis (DVT), pulmonary embolism (PE), postoperative infection, acute myocardial infarction (MI), wound dehiscence, pneumonia/pneumonitis, urinary tract infection (UTI), cerebrovascular accident (CVA), acute kidney injury (AKI), cardiogenic shock, other postoperative non-septic shock, and sepsis were used to identify these complications of interest (see Appendix A). We also used the Enhanced ICD-9 code method developed by Quan et al. to calculate a Charlson Comorbodity Index value for each inpatient record [10]. 

We used *t*-tests and the chi-square test to compare the characteristics and outcomes of octogenarians and nonagenarians undergoing PTKA and RTKA. We used binary logistic regression to compare the odds of a patient sustaining any complication, or suffering inpatient mortality between octogenarians and nonagenarians. We also compared these odds after adjusting for the following likely confounding variables: Charlson Comorbidity Index, gender, race, median household income, number of diagnoses, number of inpatient procedures, month of admission, year of admission, and whether or not admission was on a weekend. We conducted all analyses using Stata 15 (StataCorp, College Station, TX, USA) with a two-sided alpha level of 0.05. Some variables were noted to have a significant positive skew (Length of Stay, Total Charges, Number of Procedures). To determine significant differences between groups in these variables, we applied a log transformation, and then compared the means between groups with the log-transformed data. Finally, to adjust for the sampling design of the NIS dataset, we used Stata survey commands (svy) [11].

## 3. Results

A total of 65,691 hospital admissions between 2010 and 2014 were included in our study. After accounting for the sampling weights of the NIS, this represents a national estimation of 324,933 patients. Of these patients, 11,634 (3.58%) were nonagenarians, and 313,299 were octogenarians (96.42%). A total of 294,462 (90.62%) of the patients underwent PTKA, and 30,471 (9.38%) underwent RTKA.

### 3.1. Primary TKA

Nonagenarians undergoing PTKA had more chronic conditions (5.60 vs. 5.43, *p* = 0.004), number of diagnoses (9.17 vs. 8.72, *p* < 0.001), and number of procedures (Table 1). Nonagenarians and octogenarians also differed significantly in their racial distribution (*p* < 0.001) and Charlson Comorbidity Index strata distribution (*p* = 0.02). The two groups did not differ significantly in gender or median household income distribution.

Nonagenarians undergoing PTKA had a significantly longer length of stay (3.76 days vs. 3.43, *p* < 0.001) than octogenarians and significantly higher total charges ($57,956 vs. $53,651, *p* < 0.001) (Table 2). Nonagenarians also had a significantly higher inpatient mortality rate than octogenarians (1.03% vs. 0.16%, *p* < 0.001). Nonagenarians received transfusions at a significantly higher rate than octogenarians (25.93% vs. 17.88%, *p* < 0.001) and developed urinary tract infection (4.74% vs. 3.35%, *p* = 0.001), acute kidney injury (5.23% vs. 3.08%, *p* < 0.001), and sepsis (0.61% vs. 0.23%, *p* < 0.001) at a significantly higher rate. The two groups did not differ significantly in the rate of development of deep venous thrombosis, pulmonary embolism, postoperative infection, acute myocardial infarction, dehiscence, pneumonia or pneumonitis, cerebrovascular accident, cardiogenic shock, or other postoperative shock.

We performed a binary logistic regression to compare the odds of a patient sustaining any inpatient complication between octogenarians and nonagenarians undergoing PTKA. Nonagenarians had significantly higher odds of sustaining any complication (OR = 1.62, 95% CI: 1.47, 1.78). This finding was unchanged (OR = 1.65, 95% CI: 1.47, 1.86) after adjusting for likely confounding variables (see Methods section for a complete list of the likely confounding variables).

We also performed a binary logistic regression to compare the odds of a patient suffering inpatient mortality between octogenarians and nonagenarians undergoing PTKA. Nonagenarians had significantly higher odds of suffering inpatient mortality (OR = 6.29, 95% CI: 3.86, 10.24). This finding was unchanged (OR = 7.75, 95% CI: 4.41, 13.62) after adjusting for likely confounding variables. 

### 3.2. Revision TKA

Nonagenarians undergoing RTKA had more chronic conditions (6.47 vs. 5.96, *p* = 0.001), number of diagnoses (12.93 vs. 11.51, *p* < 0.001) and number of procedures, and had a higher proportion of females (70.12% vs. 58.73%, *p* < 0.001) (Table 3). Nonagenarians and octogenarians differed significantly in their distributions into the Charlson Comorbidity Index strata (*p* = 0.003). The two groups did not differ significantly in racial or median household income distribution. 

Nonagenarians undergoing RTKA had a significantly longer length of stay (5.88 days vs. 4.88, *p* < 0.001) and higher total charges ($98,828 vs. $86,203, *p* = 0.006) than octogenarians (Table 4). Nonagenarians also had a higher inpatient mortality rate (2.73% vs. 0.81%, *p* < 0.001) than octogenarians. Nonagenarians received transfusions at a rate significantly higher than octogenarians (35.33% vs. 25.38%, *p* < 0.001) and developed pneumonia or pneumonitis (1.28% vs. 0.44%, *p* = 0.020), urinary tract infection (14.06% vs. 7.20%, *p* < 0.001), acute kidney injury (15.28% vs. 8.73%, *p* < 0.001) and cardiogenic shock (0.26% vs. 0.02%, *p* = 0.012) at a significantly higher rate than octogenarians. The two groups did not differ significantly in the rate at which they developed deep venous thrombosis, pulmonary embolism, postoperative infection, acute myocardial infarction, dehiscence, cerebrovascular accident, other postoperative shock, or sepsis. 

We performed a binary logistic regression to compare the odds of a patient sustaining any inpatient complication between octogenarians and nonagenarians undergoing RTKA. Nonagenarians had significantly higher odds of sustaining any complication (OR = 1.89, 95% CI: 1.54, 2.32). This finding was unchanged (OR = 1.60, 95% CI: 1.24, 2.08) after adjusting for likely confounding variables (see Methods section for a complete list of the likely confounding variables).

We also performed a binary logistic regression to compare the odds of a patient suffering inpatient mortality between octogenarians and nonagenarians undergoing RTKA. Nonagenarians had significantly higher odds of suffering inpatient mortality (OR = 3.45, 95% CI: 1.76, 6.76). This finding was unchanged (OR = 3.22, 95% CI: 1.43, 7.28) after adjusting for likely confounding variables.

## 4. Discussion

It is estimated that by 2050, the proportion of the senior US population (age 65 and older) made up by people 90 years and older will approximately double to 10% [2]. It can be expected that as the population shifts in age distribution, so too will the composition of patients undergoing both primary and revision total knee arthroplasty. Our study employed a large nationally representative sample to search for and identify any significantly increased marginal risks in complications in this group, compared to octogenarians. 

It has been previously found that nonagenarians undergoing primary total joint arthroplasty (total knee arthroplasty and total hip arthroplasty) have a higher risk of in-hospital mortality, compared to younger patients [12]. We found that in both PTKA and RTKA, nonagenarians had a higher risk of inpatient mortality compared to octogenarians. In fact, nonagenarians had over a six times higher risk compared to octogenarians undergoing PTKA, and over a three times higher risk in patients undergoing RTKA. In addition, nonagenarians undergoing total joint arthroplasty have been found to develop urinary tract infection, and require transfusion at significantly higher rates than younger patients [8]. Our study yielded similar results. We found that nonagenarians undergoing both PTKA and RTKA had higher risks of requiring transfusion and developing urinary tract infection, as well as developing acute kidney injury compared to octogenarians.

Nonagenarians undergoing both PTKA and RTKA had a significantly longer length of stay and significantly higher total charges than octogenarians, which aligns with previous findings [3,12]. These outcomes are likely explained by the significantly increased risk of developing certain complications. The additional time and resources needed to care for these complications likely accounts for the increased length of stay, and the total charges of nonagenarians, compared to octogenarians.

In recent years, interest in bundled payment models, particularly in the context of total joint arthroplasty, has grown. These models, in which one lump sum payment is exchanged for an entire episode of care, have the potential to lower healthcare spending [13]. The economic viability of these models is contingent upon the model’s ability to accurately and reliably predict the cost of a patient’s episode of care. Our findings suggest that age is an important parameter that must be included when attempting to predict the complication rate and ultimate cost of a patient’s episode of care. 

In our study, nonagenarians undergoing both PTKA and RTKA had a higher number of diagnoses and chronic conditions than octogenarians, as has been previously observed [3]. Furthermore, in both PTKA and RTKA, we found that nonagenarians and octogenarians differed significantly in the Charlson Comorbidity Index. However, we found that, in both primary and revision TKA, nonagenarians were significantly more likely to sustain both inpatient complications and mortality, compared to octogenarians, even when controlling for likely confounding variables, including the Charlson Comorbidity Index. Therefore, age may be an independent risk factor for inpatient complications and inpatient mortality. Further research in this area should focus on confirming or refuting this finding, and, if this finding holds, assessing the relative weight of age as an independent variable that predicts inpatient complication rate and mortality risk.

TKA offers patients the potential for significant pain reduction, and an improved quality of life [14]. In particular, patients aged 80–95 have been found to have a much higher rate of “complete relief” of pain with TKA, compared to patients aged 65–69 [15]. Furthermore, increased age appears to be associated with a greater improvement in the patient-reported quality of life scores, one year following surgery [14]. This suggests that octogenarians and nonagenarians in particular, stand to benefit markedly from TKA. However, these benefits must be weighed against the potential risks of surgical complications. 

In both primary and revision TKA, inpatient mortality rate is significantly higher in nonagenarians than octogenarians (RTKA: 2.73% vs. 0.81%; PTKA: 1.03% vs. 0.16%). This finding should be put into the context of general life expectancy trends by age group. The Social Security Administration (SSA) publishes data on the “Death Probability”, or likelihood of someone dying within the next year, of Americans of all ages. This data illustrates how there is likely a major difference in the baseline short-term mortality rate between our two cohorts. For example, we can compare the Death Probability of people with the mean age of our octogenarian cohort (about 83) and nonagenarian cohort (about 91). While an 83-year-old male has an 8.03% chance of dying in the next year, and an 83-year-old female has a 6.04% chance, a 91-year-old male has an 18.4% chance, and a 91-year-old female has a 14.8% chance [16]. 

Our study is limited primarily by the applicability of the ICD-9 codes that we used. We used ICD-9 codes to identify our treatment groups (primary and revision total knee arthroplasty procedures) and assess for inpatient complications. We used codes previously described to identify patients undergoing PTKA and RTKA [17]. We did not include ICD-9 code 80.06 (arthrotomy for the removal of prosthesis without replacement, knee) though it has been used previously to identify RTKA [18]. We felt that excluding this code improved our specificity, though we acknowledge that it may have also reduced our sensitivity in detecting RTKA. To identify complications, we used a combination of codes previously described (as described by Vogel et al. in the case of sepsis) as well as our own investigation to determine the most applicable ICD-9 codes [19]. We recognize that there may be other codes that would better capture the complications of interest with improved sensitivity and/or specificity.

In addition, by having an exceptionally large sample size, our study is susceptible to committing Type I errors. With such a large *n* value, even minute differences in complication rates may be statistically significant at a 5% alpha level. Therefore, it is important to note that statistically significant differences in complication rates between octogenarians and nonagenarians should also be evaluated for clinical relevance. For example, while nonagenarians sustained pneumonia or pneumonitis at a rate that was statistically significantly higher than octogenarians, the *p*-value (0.02) of this finding is not nearly as low as many of the other differences that we observed, and the actual difference in complication rate is relatively small (0.44% in octogenarians vs. 1.28% in nonagenarians). This is an instance where further research would be needed to determine the clinical relevance of this statistically significant difference in complication rate.

The strengths of our study include our use of a large nationally representative dataset. We included over 300,000 patients in our study, which lends high precision to our findings. In addition, because the NIS is nationally representative, our study has wide generalizability.

## 5. Conclusions

In conclusion, our study suggests that orthopedic surgeons can expect increased rates of some complications in nonagenarians undergoing both PTKA and RTKA, compared to octogenarians. While the bulk of these complications are minor, nonagenarians do experience postoperative inpatient mortality at a significantly higher rate than octogenarians, following both procedures. Our study can help inform the expectations and guide the inpatient postoperative management of orthopedic surgeons operating on nonagenarians, to help mitigate some of the increased risk of select postoperative complications, and to thereby help to reduce length of stay and total charges. Future research should focus on evaluating the clinical relevance of the differences in complication rates that we observed, as well as incorporating patient-reported outcomes. Moving forward, our study can serve as a reference for orthopedic surgeons and primary care providers to use when discussing postoperative expectations and rates of specific complications in patients of both age groups. Our findings can assist doctors and patients in working together to determine if the expected benefits of total knee arthroplasty are worth the risks.

## Figures and Tables

**Table 1:** Comparing characteristics of patients undergoing primary total knee arthroplasty between octogenarians and nonagenarians. Healthcare Cost and Utilization Project—Nationwide Inpatient Sample, 2010–2014.

Patient Characteristics	Octogenarians	Nonagenarians	*p* Value
Age (years), mean (SD)	82.91 (2.42)	90.53 (1.24)	-
Number of Chronic Conditions, mean (SD)	5.43 (2.50)	5.60 (2.55)	0.004
Number of Diagnoses, mean (SD)	8.72 (4.39)	9.17 (4.55)	<0.001
Number of Procedures, mean (SD)	1.66 (1.02)	1.74 (1.06)	<0.001 *
Female	64.10%	65.94%	0.094
Race			<0.001
White	87.57%	90.90%	
Black	4.24%	2.81%	
Hispanic	4.49%	3.10%	
Asian or Pacific Islander	1.36%	1.00%	
Native American	0.39%	0.11%	
Other	1.94%	2.08%	
Median Household Income (1 = lowest quartile, 4 = highest quartile)			0.422
1	21.24%	21.44%	
2	27.17%	28.09%	
3	26.75%	25.06%	
4	24.84%	25.41%	
Charlson Comorbidity Index			0.02
4	52.59%	52.30%	
5	25.96%	23.93%	
6+	21.45%	23.77%	

* *t*-test performed on the log-transformed data to meet the assumption of normality.

**Table 2:** Comparing the outcomes and complications of patients undergoing primary total knee arthroplasty between octogenarians and nonagenarians. Healthcare Cost and Utilization Project—Nationwide Inpatient Sample, 2010–2014.

Patient Outcomes	Octogenarians	Nonagenarians	*p*-Value
Length of Stay (days), mean (SD)	3.43 (2.04)	3.76 (2.41)	<0.001 *
Total Charges (USD), mean (SD)	53,651 (34,250)	57,956 (41,253)	<0.001 *
% Hospital Mortality	0.16%	1.03%	<0.001
% Receiving Transfusion	17.88%	25.93%	<0.001
% Deep Venous Thrombosis (DVT)	0.22%	0.05%	0.091
% Pulmonary Embolism (PE)	0.43%	0.41%	0.887
% Postop Infection	0.13%	0.19%	0.402
% Acute Myocardial Infarction (MI)	0.40%	0.62%	0.145
% Dehiscence	0.07%	0.05%	0.815
% Pneumonia or Pneumonitis	0.51%	0.36%	0.350
% Urinary Tract Infection (UTI)	3.35%	4.74%	<0.001
% Cerebrovascular Accident (CVA)	0.08%	0.10%	0.723
% Acute Kidney Injury (AKI)	3.08%	5.23%	<0.001
% Cardiogenic Shock	0.004%	0.00%	0.794
% Other Shock	0.05%	0.09%	0.464
% Sepsis	0.23%	0.61%	<0.001

* *t*-test performed on log-transformed data to meet the assumption of normality.

**Table 3:** Comparing characteristics of patients undergoing revision total knee arthroplasty between octogenarians and nonagenarians. Healthcare Cost and Utilization Project—Nationwide Inpatient Sample, 2010–2014.

Patient Characteristics	Octogenarians	Nonagenarians	*p* Value
Age (years), mean (SD)	83.21 (2.54)	90.58 (1.34)	-
Number of Chronic Conditions, mean (SD)	5.96 (2.83)	6.47 (2.91)	0.001
Number of Diagnoses, mean (SD)	11.51 (5.26)	12.93 (5.61)	<0.001
Number of Procedures, mean (SD)	2.68 (1.83)	2.91 (1.97)	0.006 *
Female	58.73%	70.12%	<0.001
Race			0.376
White	86.72%	88.95%	
Black	6.19%	4.18%	
Hispanic	3.63%	4.07%	
Asian or Pacific Islander	0.86%	1.36%	
Native American	0.45%	0.00%	
Other	2.14%	1.45%	
Median Household Income (1 = lowest quartile, 4 = highest quartile)			0.656
1	22.57%	23.85%	
2	28.18%	26.05%	
3	26.06%	25.02%	
4	23.19%	25.08%	
Charlson Comorbidity Index			0.003
4	42.78%	37.41%	
5	27.45%	24.58%	
6+	29.78%	38.01%	

* *t*-test performed on log-transformed data to meet the assumption of normality.

**Table 4:** Comparing outcomes and complications of patients undergoing revision total knee arthroplasty between octogenarians and nonagenarians. Healthcare Cost and Utilization Project—Nationwide Inpatient Sample, 2010–2014.

Patient Outcomes	Octogenarians	Nonagenarians	*p*-Value
Length of Stay (days), mean (SD)	4.88 (4.64)	5.88 (5.07)	<0.001 *
Total Charges (USD), mean (SD)	86,203 (70,541)	98,828 (74,803)	0.006 *
% Hospital Mortality	0.81%	2.73%	<0.001
% Receiving Transfusion	25.38%	35.33%	<0.001
% DVT	0.66%	0.49%	0.678
% PE	0.66%	0.26%	0.331
% Postop Infection	0.68%	1.23%	0.197
% Acute MI	0.73%	1.28%	0.226
% Dehiscence	1.38%	1.96%	0.338
% Pneumonia or Pneumonitis	0.44%	1.28%	0.020
% UTI	7.20%	14.06%	<0.001
% CVA	0.10%	0.33%	0.247
% AKI	8.73%	15.28%	<0.001
% Cardiogenic Shock	0.02%	0.26%	0.012
% Other Shock	0.36%	0.00%	0.228
% Sepsis	3.83%	3.83%	0.998

* *t*-test performed on log-transformed data to meet the assumption of normality.

## Appendix A

**Table A1:** ICD-9 Codes Used to Identify Postoperative Complications.

Complication	ICD-9 Code	ICD-9 Code Description
Transfusion	99.03	Other transfusion of whole blood
	99.04	Transfusion of packed cells
Acute Deep Venous Thrombosis of Lower Extremity	453.40	Acute venous embolism and thrombosis of unspecified deep vessels of lower extremity
	453.41	Acute venous embolism and thrombosis of deep vessels of proximal lower extremity
	453.42	Acute venous embolism and thrombosis of deep vessels of distal lower extremity
Pulmonary Embolism	415.1	Pulmonary embolism and infarction
	415.11	Iatrogenic pulmonary embolus and infarction
	415.13	Saddle embolus of pulmonary artery
	415.19	Other pulmonary embolism and infarction
Postoperative Infection	998.59	Other postoperative infection
	998.51	Infected postoperative seroma
	998.5	Postoperative infection not elsewhere classifed
Acute Myocardial Infarction	410.01	Acute myocardial infarction of anterolateral wall, initial episode of care
	410.11	Acute myocardial infarction of other anterior wall, initial episode of care
	410.21	Acute myocardial infarction of inferolateral wall, initial episode of care
	410.31	Acute myocardial infarction of inferoposterior wall, initial episode of care
	410.41	Acute myocardial infarction of other inferior wall, initial episode of care
	410.51	True posterior wall infarction, initial episode of care
	410.61	Acute myocardial infarction of anterolateral wall, initial episode of care
	410.71	Subendocardial infarction, initial episode of care
	410.81	Acute myocardial infarction of other specified sites, initial episode of care
	410.91	Acute myocardial infarction of unspecified site, initial episode of care
Dehiscence	998.30	Disruption of wound, unspecified
	998.31	Dehiscence of internal operation wound
	998.32	Dehiscence of external operation wound
Pneumonia or Pneumonitis	997.39	Postoperative pneumonia or postoperative pneumonitis
	997.32	Postoperative pneumonia or postoperative pneumonitis, aspiration type
Urinary Tract Infection	599.0	Urinary tract infection, site not specified
Cerebrovascular Accident	997.02	Postoperative cerebrovascular accident
Acute Kidney Failure	584	Acute kidney failure
	584.5	Acute kidney failure with lesion of tubular necrosis
	584.6	Acute kidney failure with lesion of renal cortical necrosis
	584.7	Acute kidney failure with lesion of renal medullary (papillary) necrosis
	584.8	Acute kidney failure with other specified pathological lesion of kidney
	584.9	Acute kidney failure, unspecified
Cardiogenic Shock	998.01	Postoperative cardiogenic shock
Other Postoperative Shock	998.0	Postoperative shock not elsewhere specified
	998.00	Postoperative shock, unspecified
	998.09	Postoperative shock, other
Sepsis	998.02	Postoperative septic shock
	038.0	Streptococcal septicemia
	038.10	Staphylococcal septicemia unspecified
	038.11	*Staphylococcus aureus* septicemia
	038.19	Other staphylococcal septicemia
	038.2	Pneumococcal septicemia
	038.3	Septicemia due to anaerobes
	038.40	Septicemia due to Gram-negative organism unspecified
	038.41	Septicemia due to *Hemophilus influenza*
	038.42	Septicemia due to *Escherichia coli*
	038.43	Septicemia due to *Pseudomonas*
	038.44	Septicemia due to *Serratia*
	038.49	Septicemia due to other Gram-negative organisms
	038.8	Other unspecified septicemias
	038.9	Unspecified septicemia
	785.52	Septic shock
	785.59	Other shock without mention of trauma
	995.91	Systemic inflammatory response syndrome due to infectious process without organ dysfunction
	995.92	Systemic inflammatory response syndrome due to infectious process with organ dysfunction
